# Theoretical Investigation of Biaxially Tensile-Strained Germanium Nanowires

**DOI:** 10.1186/s11671-017-2243-1

**Published:** 2017-07-28

**Authors:** Zhongyunshen Zhu, Yuxin Song, Qimiao Chen, Zhenpu Zhang, Liyao Zhang, Yaoyao Li, Shumin Wang

**Affiliations:** 1 State Key Laboratory of Functional Materials for Informatics, Shanghai Institute of Microsystem and Information Technology, Chinese Academy of Sciences, Shanghai, 200050 China; 2 School of Physical Science and Technology, ShanghaiTech University, Shanghai, 201210 China; 3 University of Chinese Academy of Sciences, Beijing, 100190 China; 4 Department of Microtechnology and Nanoscience, Chalmers University of Technology, Gothenburg, 41296 Sweden

**Keywords:** Tensile strain, Ge nanowire, Finite element method, Direct bandgap, Mobility

## Abstract

We theoretically investigate highly tensile-strained Ge nanowires laterally on GaSb. Finite element method has been used to simulate the residual elastic strain in the Ge nanowire. The total energy increment including strain energy, surface energy, and edge energy before and after Ge deposition is calculated in different situations. The result indicates that the Ge nanowire on GaSb is apt to grow along 〈100〉 rather than 〈110〉 in the two situations and prefers to be exposed by {105} facets when deposited a small amount of Ge but to be exposed by {110} when the amount of Ge exceeds a critical value. Furthermore, the conduction band minima in *Γ*-valley at any position in both situations exhibits lower values than those in L-valley, leading to direct bandgap transition in Ge nanowire. For the valence band, the light hole band maxima at *Γ*-point is higher than the heavy hole band maxima at any position and even higher than the conduction band minima for the hydrostatic strain more than ∼5.0%, leading to a negative bandgap. In addition, both electron and hole mobility can be enhanced by owing to the decrease of the effective mass under highly tensile strain. The results suggest that biaxially tensile-strained Ge nanowires hold promising properties in device applications.

## Background

As a group IV element, germanium (Ge) holds the superiority both in optoelectronics and electronics and has great compatibility with silicon (Si) photonics. An essential characteristic of Ge is that its direct bandgap is around 0.8 eV (1.55 *μ*m) at 300 K. The mobility of both electron and hole in Ge is much higher than that in Si. Thus, Ge has been utilized in high speed devices in current Si-based integrated circuit [1, 2]. More interestingly, tensile-strained Ge offers optimization in the above aspects. Ge is an indirect bandgap semiconductor with a slight difference of 136 meV between L-valley and *Γ*-valley [3]. It is theoretically predicted that over ∼4.0% uniaxial along 〈111〉 [4] or ∼1.6–2.0% biaxial [5, 6] tensile strain can lower the *Γ*-valley below the L-valley, thereby converting Ge into a direct bandgap material, which opens a new route for light emitting from group IV materials. Another important point is that tensile-strained Ge provides significant enhancement in carrier mobility [7, 8] for realizing high speed complementary metal oxide semiconductor devices.

Nanowires (NWs) exhibit attractive electronic and optical properties owing to the large surface to volume ratio and confinement of both carriers and photons in two dimensions (2D) [9]. In the past years, Ge NWs (GeNWs) on Si [10] or Ge/Si core/shell NWs [11] have been under extensive study due to their potentials in metal oxide semiconductor field-effect transistors (MOSFETs) for Si microelectronics. Both the scaled transconductance and on-current were enhanced by three to four times compared with conventional Si *p*-MOSFET [12]. Thus, epitaxial growth of lateral GeNWs directly on Si has been expected to fabricate high performance MOSFETs. Zhang et al. addressed that ultrathin GeNWs on Si (001) are exposed with facets of {105} [13], which have the lowest predicted surface energy [14]. Further, strained Ge MOSFET on SiGe virtual substrate was shown to improve the hole mobility [1]. Although lateral GeNW on Si with compressive strain can display significant improvement in carrier transport as assumed, they are unable to be converted into a direct bandgap. For introducing tensile strain in GeNWs, GeSn alloy [15] and III-Sb compounds [16], which have a larger lattice constant than that of Ge, are required. In spite of that mechanical method has been applied for fabricating uniaxially strained GeNWs [17, 18], the complex of this fabrication technique can be hardly suitable for monolithic integration in Si-based photonics and electronics. Additionally, the strain will release easily in free-standing NWs, whereas lateral GeNWs can accommodate much high strain in themselves. Thus, epitaxially grown lateral GeNWs with high biaxial tensile strain are required for achieving direct bandgap transition as well as carrier mobility enhancement.

To date, dislocation-free and highly biaxial tensile-strained Ge quantum dots on InP (001) have shown potentials for direct bandgap emitting simulated by finite element method (FEM) [19]. Similar to this, in this work, we theoretically predict morphology of exposed surfaces and growth direction of biaxially tensile-strained GeNWs on a relaxed GaSb template that can be grown directly on Si with an AlSb buffer layer [16, 20]. We choose {110}, {105}, and {111} as exposed surfaces of lateral GeNWs and compare the total energy change in the steady-state system. We ignore the influence of dislocation and fracture [21] in this highly strained system for simplicity. The simulation based on FEM reveals that there exists a critical amount of Ge. Below the critical value, GeNWs are exposed by {105}, while above the critical value, they are exposed by {110}. Almost all the GeNW region can be converted into direct bandgap, which is the difference of the conduction band minima and the light hole band maxima at the *Γ*-point. Besides, we also qualitatively analyze the change of strain-dependent carrier effective mass at the *Γ*-point to indirectly predict the enhancement in both electron and hole mobility.

## Methods

**Fig. 1:**
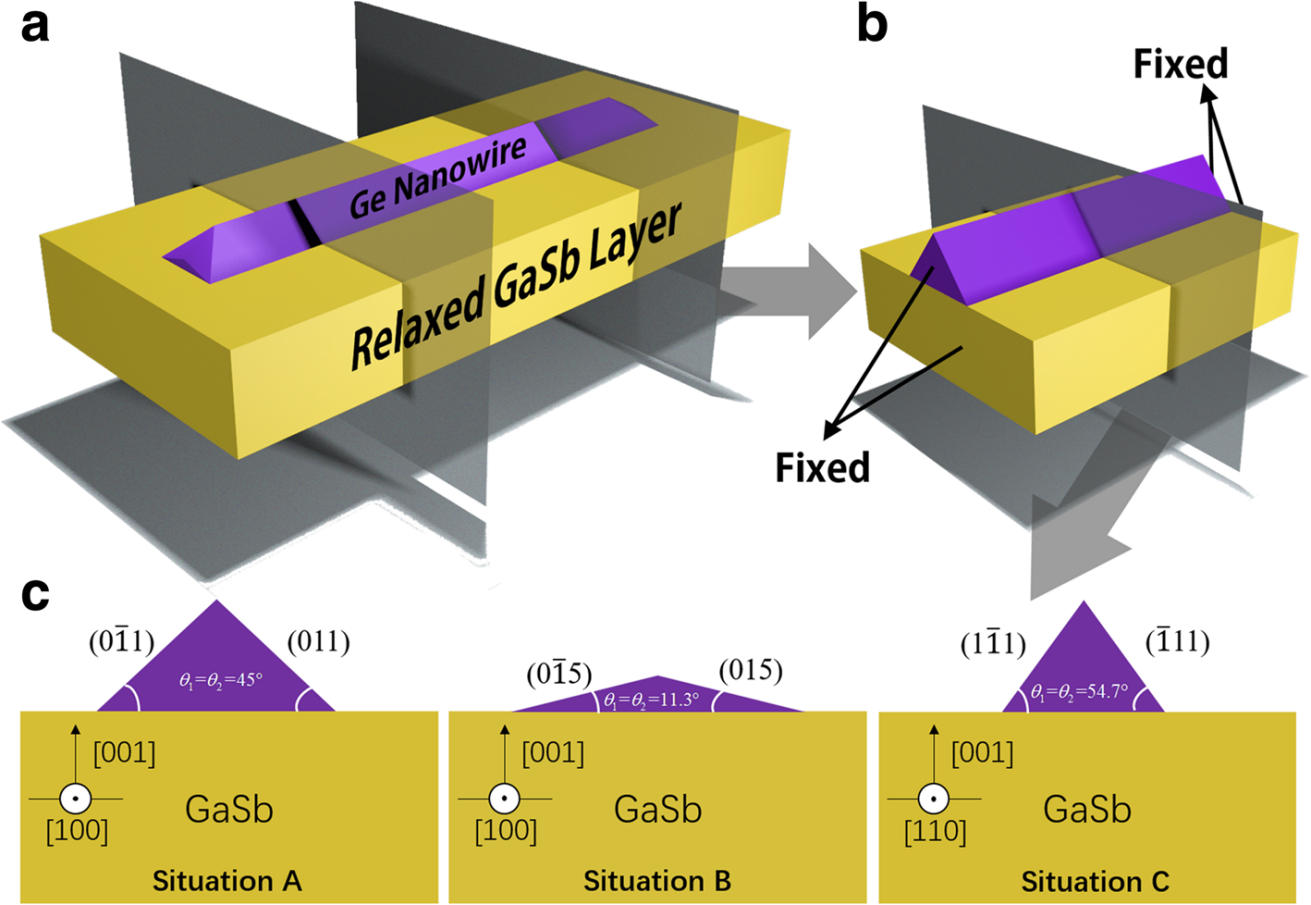
Schematics of GeNW on GaSb: **a** 3D model, **b** simplified finite model, and **c** cross sections of GeNW with different facets

Lateral NWs normally reveal a triangle shape of cross section [22, 23]. Thanks to the 1D property of NW shown in Fig. 1a, an finite NW model similar to Zhang et al. in the supplemental material of Ref. [13] can be properly used for simulation in which both beginning and ending surfaces are fixed shown in Fig. 1b. Because of the boundary effect, we only discuss the center part of the NW and consider that the cross section of this part represents the situation in an infinitively long NW. FEM is used to simulate distribution of tensile strain in GeNW with 7.7% lattice mismatch to GaSb. We calculate the total system energy increment after depositing the same amount of Ge in steady state in three situations: (i) [100] growth direction with {110} facets exposed (situation A), (ii) [100] growth direction but with {105} facets exposed (situation B), and (iii) [110] growth direction with {111} facets exposed (situation C). The cross sections of these three situations are shown in Fig. 1c. The areas of cross section are kept the same, representing equal amounts of Ge.

The total system energy change per unit length (J/nm) includes overall difference in strain energy, surface energy, and edge energy [24] and can be given by:

1 $$ \Delta{{E}_{\text{total}}}=\Delta{{E}_{\text{strain}}}+\Delta{{E}_{\text{surface}}}+\Delta{{E}_{\text{edge}}}.  $$

The first term *Δ* *E*_*strain*_ represents the strain energy difference of the steady system before and after Ge deposition and is expressed in unit volume,

2 $$ \Delta{u}=\frac{1}{2{Y}}\sum\limits_{i=j}^{{}}{\tau_{ij}^{2}}-\frac{\nu }{Y}\sum\limits_{i<j}^{{}}{{{\tau }_{ii}}{{\tau }_{jj}}}+\frac{1}{2{G}}\sum\limits_{i<j}^{{}}{\tau_{ij}^{2}}  $$

where *τ*_*ij*_=*C*_*ij*_ *ε*_*ij*_ (*i, j*=1, 2, 3) denote the stress tensor, *C*_*ij*_ and *ε*_*ij*_ are the elastic constants and strain tensor, respectively, *Y* and *G* are the equivalent Young’s modulus and the equivalent shear modulus, respectively, and *ν* is the equivalent Poisson ratio. In the 2D cross section considered, the total strain energy change can be obtained by integrating area with the value of *Δ* *u* on every point. The second term *Δ* *E*_*surface*_ is related to the difference of surface energy before and after Ge deposition. The surface energy in the cross section of our situations can be rewritten as [23]

3 $$ {{\gamma}_{i}}=\frac{2h}{\sin{{\theta}_{i}}}({{\sigma}_{i}}-{{\sigma}_{{(001)}}}\cos{{\theta}_{i}})  $$

where *h* is the height of the GeNW cross section, *σ*_*i*_ (*i*=A, B, and C) is the average surface energy of the exposed facets under unit area, and the corresponding angel between the NW facets and interface is labeled by *θ*_*i*_ as schematically shown in Fig. 1c. The strain in surface energy can be neglected due to its slight impact as the treatment in Ref. [25]. Table 1 shows surface energy values from literature. The last term called edge energy change stands for the change of energy cost for forming sharp edges and is given by

4 $$ \Delta{{E}_{\text{edge}}}=3\varGamma  $$

**Table 1:** The average surface energy of different facets in Ge and GaSb at 300 K

Material	Facets	Surface Energy (eV/nm^2^)	Reference
Ge	{110}	8.125^a^	[34]
	{105}	6.4^b^	[14]
	{111}	6.625^a^	[34]
GaSb	{100}	10^a^	[35]

^a^The value from experiments

^b^Theoretical results

where 3Γ is the total edge energy containing the top and two basal facet intersections. The estimated value of 3Γ is 3.7 eV/nm by experimental fitting [26] and the influence of the edge energy can be ignored due to the hardly varied value [25]. Hence, it is valid to calculate the energy increment of *Δ* *E*_total_−3Γ.

After knowing the strain distribution, the strain-dependent conduction band decrement at the *Γ*- and the L-valley can be calculated with neglecting the quantum effect. The conduction band of the *Γ*-valley is lowered only with hydrostatic strain by

5 $$ \Delta E_{c}^{\Gamma}={{a}_{c}}({{\varepsilon}_{xx}}+{{\varepsilon}_{yy}}+{{\varepsilon}_{zz}})  $$

where *a*_*c*_ denotes hydrostatic deformation potential with the value of −8.24 eV at the *Γ*-point [27], *ε*_*xx*_, *ε*_*yy*_ and *ε*_*zz*_ are the strain in *x*, *y,* and *z* direction in the material coordinate system, respectively. However, the shift of the conduction band at the L-point is subject to both hydrostatic and shear strain [19], given by

6 $$ {{}\begin{aligned} \Delta{E_{c}^{\mathrm{L}}}=&\left({{\Xi}_{d}}+\frac{1}{3}{{\Xi}_{u}}\right)({{\varepsilon}_{xx}}+{{\varepsilon}_{yy}}+{{\varepsilon}_{zz}})\\&-\frac{2}{3}{{\Xi}_{u}}\left(|{{\varepsilon}_{xy}}|+|{{\varepsilon}_{yz}}|+|{{\varepsilon}_{xz}}|\right) \end{aligned}}  $$

where *Ξ*_*d*_ and *Ξ*_*u*_ are dilation deformation potential with the value of −6.97 eV and uniaxial deformation potential with the value of 16.3 eV at the L-valley, respectively. For converting Ge into a direct bandgap material, the *Γ*-valley has to be lower than the L-valley, which means $\Delta {E_{c}^{\Gamma, \text {L}}}=\Delta E_{c}^{\Gamma }-\Delta {E_{c}^{\mathrm {L}}}<-0.136$ eV. Here, we use $\Delta {E_{\text {DT}}}=\Delta E_{c}^{\Gamma, \text {L}}+0.136$ to present the difference in the *Γ*- and the L-point. Once the *Γ*-point descends below the L-point, *Δ* *E*_DT_ will be negative. A series of tensile-strained GeNWs with varied sizes are simulated to show the direct bandgap transition.

In addition, due to the high tensile strain in the GeNW, the *Γ*-valley is below the L-valley while the light hole band maxima becomes the valence maxima [28]. The bandgap in such a high tensile-strained GeNW will be the difference between the *Γ*-valley and the light hole band maxima at the *Γ*-point. Thus, the spatially distributed bandgap as well as the band edge energies at the *Γ*-point which is **k**=0 including conduction band, heavy hole band, and light hole band are calculated by eight-band **k.p** theory [29]. We ignore the quantum effect since it is very weak in our GeNW model with a 40-nm basal width. The result can be applied to study the electron-hole recombination in the tensile-strained GeNW as well as the mechanism of mobility enhancement. Generally, the electron or hole mobility can be given by *μ*=*e* *τ*/*m* ^∗^, where *m* ^∗^ is the carrier effective mass and *τ* is the electron-phonon scattering time. In the model of a parabolic approximation for the *Γ*- and L-valleys with isotropic scattering, the scattering time is proportional to $m_{DOS}^{*-3/2}$, leading to the conclusion that the mobility ratio reaches *μ*_*Γ*_/*μ*_L_=182 if the *Γ*-valley moves below the L-valley and both the electron-phonon scattering time and the effective mass of electrons are invariable with strain [30]. However, with the consideration of the complexity in the calculation of anisotropic scattering and strain-dependent effective mass in our NW model, we only qualitatively analyze the improvement in both electron and hole mobility in a highly tensile-strained GeNW via the decrease of both electron and hole effective mass at the *Γ*-point.

## Results and Discussions

**Fig. 2:**
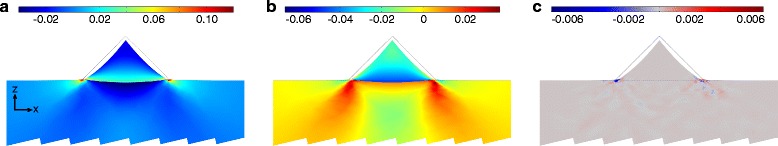
Residual strain distribution of a GeNW in situation A with the basal width of 40 nm: **a** x component strain *ε*_*xx*_, **b** z component strain *ε*_*zz*_, and **c** shear strain in x-y plane *ε*_*xy*_. The zigzag shape at the bottom denotes the partial substrate layer (the following has the same meaning)

We consider the system is initially under full tensile strain owing to the large lattice mismatch of 7.7% between Ge and GaSb. Figure 2 shows the 2D residual strain distribution including in-plane strain *ε*_*xx*_, shear strain *ε*_*xy*_, and vertical strain *ε*_*zz*_ of situation A with the base width of *w* = 40 nm for example in the steady state after relaxation. The strain definition here is (*a*_Ges_−*a*_Ge_)/*a*_Ge_, where *a*_Ges_ and *a*_Ge_ are lattice constants of strained and relaxed Ge, respectively. As seen in Fig. 2a, *ε*_*xx*_ has the maximum value of about 15.4% at two basal edges which is much larger than the initial strain, but decreases sharply from the edge to the center with the minimum value of about 3.3%. In *z*-direction from the bottom to the top of GeNW, *ε*_*xx*_ also drops due to the relaxation of GeNW. The distribution of *ε*_*zz*_ is found to have similar characteristics with *ε*_*xx*_ in Fig. 2b. Unlike biaxial strain in Ge thin film, Fig. 2c shows that the asymmetrically distributed shear strain component of GeNW plays a significant role in direct bandgap transition. The strain distribution is quite similar during three situations. Nevertheless, the values of strain components are different in three situations because of the diverse width-height ratio (*W*/*H*) induced by its shape. Situation B with GeNW exposed by {105} surfaces has the largest *W*/*H* of 10, exhibiting high strain similar to that in the Ge thin film. Situation C with {111} surfaces exposed shows high strain as well since the NW growth orientation along the [110] remains an invariable value of strain, raising the value of *ε*_*xx*_ and *ε*_*yy*_ simultaneously. Thus, the in-plane strain can hardly be relaxed.

**Fig. 3:**
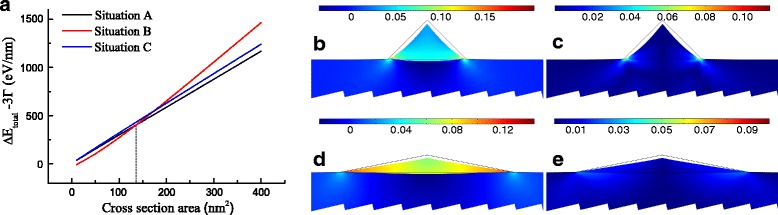
**a** The total energy change excluding the edge energy change 3 *Γ* vs. the cross section area (the *dash line* is the critical value of minima about 136.2 nm^2^ in different situations). **b**–**e** hydrostatic strain and |*ε*_*xy*_|+|*ε*_*yz*_|+|*ε*_*xz*_| in **b**, **c** situation A and **d**, **e** situation B at the critical value

In accordance with the strain distribution, the strain energy increment can be obtained. As discussed previously, situation B holds the highest strain energy increment, while situation A has the lowest one. However, for the surface energy change, situation B gives decreasing negative values with increasing area of cross section and the other two situations reveal very close positive values under the same area. The total energy increment excluding edge energy change is shown in Fig. 3a. The result shows that it is less likely to form GeNWs only in situation C due to the energy increment never being the lowest. There are two different consequences of the energy increment with the increase of the area, and the vertical dash line is marked to present the critical value of area, A_*c*_=136.2nm^2^, which means the amounts of Ge. When the area is less than 136.2 nm^2^, GeNWs are inclined to form the shape in situation B, but in situation A after depositing more Ge. The calculation result predicts that tensile-strained GeNWs on GaSb may prefer to forming high *W*/*H* triangle shape in cross section when a low amount of Ge is deposited, whereas forming low *W*/*H* one after exceeding the critical value. Figure 3b–e shows the distributions of hydrostatic strain and the sum of absolute value of shear strain components in situation A and B under the critical area. Comparing situation A with B, in spite of that situation A holds the greater maximum value of both hydrostatic strain and |*ε*_*xy*_|+|*ε*_*yz*_|+|*ε*_*xz*_|, situation B has larger average hydrostatic strain but smaller average value of |*ε*_*xy*_|+|*ε*_*yz*_|+|*ε*_*xz*_|. Meanwhile, situation B demonstrates a small difference in spatial distribution of both hydrostatic strain and |*ε*_*xy*_|+|*ε*_*yz*_|+|*ε*_*xz*_|. These properties are very similar to Ge thin film and are attributed to its high *W*/*H* value. As a result, referring to Eqs. (5) and (6), situation B exhibits smaller value of *Δ* *E*_DT_ than that of situation A, leading to a high possibility to convert Ge into a direct bandgap material.

**Fig. 4:**
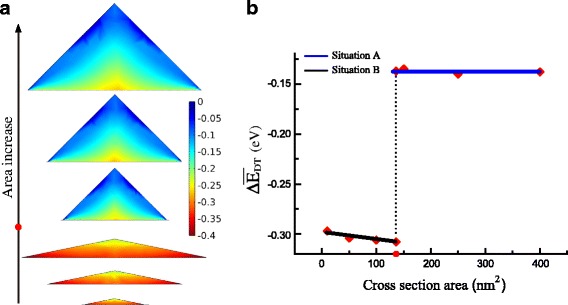
**a** The distribution of *Δ* *E*_DT_ with increase of the area (the *color bar* denotes the value of *Δ* *E*_DT_). **b** The average of *Δ* *E*_DT_ vs. the area. The critical value is marked in *red dot* in the area axis

**Fig. 5:**
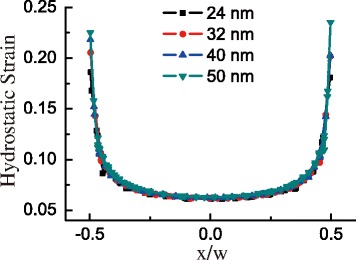
Hydrostatic strain component at the base of GeNW vs. x/w with different basal widths

Because of the high tensile strain in GeNW, nearly all the region of GeNW can be converted into direct bandgap. Figure 4a shows the distribution of *Δ* *E*_DT_ with increasing the size of GeNW. The value of *Δ* *E*_DT_ drops from the top to the bottom in GeNWs. Interestingly, the minimum of *Δ* *E*_DT_ is located in the bottom edge of the cross section in situation B, but in the bottom center in situation A. The reason of this different distribution is that the significant shear strain at the bottom edge in situation A contributes more to $\Delta E_{c}^{\mathrm {L}}$ than that in situation B. For GeNW in situation B below the critical area, the average *Δ* *E*_DT_ is much lower than that in situation A above the critical area as shown in Fig. 4b. At the critical point, the average of *Δ* *E*_DT_ suddenly rises from the value of −0.308 to −0.137 eV. Furthermore, for the same shape, the distribution and the average of *Δ* *E*_DT_ are basically similar, without obvious relation to the size. In order to discover the inherent relation to the strain in GeNW, we plot the hydrostatic strain component at the GeNW base with basal width in Fig. 5. The curves of hydrostatic strain component with different sizes versus the relative position of the base in a GeNW almost overlap except for the difference in the peripheral region. The consistent consequences are found in *ε*_*xx*_ and the sum of absolute shear components. Thus, the strain-induced value of *Δ* *E*_DT_ possesses the identical distribution in GeNW with the same shape.

**Fig. 6:**
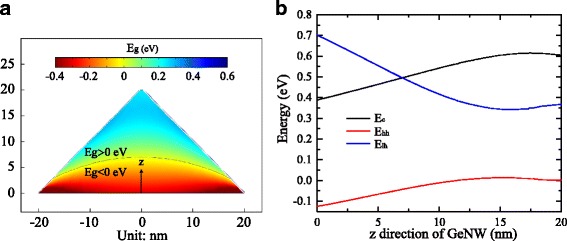
**a** The strain-dependent bandgap in the GeNW distributed by positions. **b** The strain-dependent band edge energies along *z* direction in the GeNW

Further, we simulate the strain-induced bandgap mapping in the cross section of the 40-nm wide GeNW shown in Fig. 6a. The spatial distribution of bandgap is derived from the variable value of strain-dependent band edges at the *Γ*-point. Figure 6b exhibits the band edge energy at *Γ*-point along the *z*-direction of the GeNW. We find that both the conduction band and the valence band edges including the light hole band and the heavy hole band change significantly in the first 15 nm and then slightly. The tops of the light and the heavy hole band separate and tend to shift in opposite directions with increasing the tensile strain. From Fig. 6a, b, the bandgap significantly increases in the first 15 nm to reach about 0.30 eV, then changes slightly around a value of 0.24 eV, which is the bandgap in the most GeNW regions. Since the light hole band maxima is higher than that of the heavy hole band at the *Γ*-point in the GeNW, holes in the valence band prefer to situating at the light hole band maxima. Thus, the electron-hole recombination will occur between the conduction band minimum and the light hole band maximum at the *Γ*-point if we neglect the overlap of the space-dependent electron and hole wave functions. Interestingly, the light hole band maxima moves even above the conduction band minima in the region of the NW bottom marked as a black curve in Fig. 6a with hydrostatic strain more than ∼5.0%. The negative bandgap we calculate under highly tensile strain may cause complicated consequences such as semimetallic [31] or inverted [32] band structure.

Finally, the mobility of electrons and holes will be enhanced under such highly tensile strain in the GeNW. For relaxed Ge, the electron transport is mainly contributed by electrons domiciled in the L-valley. When Ge is under tensile strain so that the *Γ*-valley is below the L-valley as shown in Fig. 3a, the primary contribution of electron transport is from the *Γ*-valley. On the other hand, the dominant participation of holes in the transport is from the light hole band at the *Γ*-point under highly tensile strain, while the heavy hole band occupies the valence band maxima in the relaxed case. Due to much smaller electron effective mass at the *Γ*-point than that at the L-point, as well as the decreased effective mass from the heavy hole band maxima to the light hole band maxima, the mobility of not only electrons but also holes can be enhanced. The tensile strain can be theoretically predicted to reduce the effective mass of electrons and holes at the *Γ*-point in a model of quantum dot by *Califano* and *Harrison* [29]. Although the quantitative calculation method is unsuitable for our NW model, we qualitatively assume that tensile strain can modify the effective mass at **k**=0 by increasing the curvature of dispersion relation for small **k** in the vicinity of the *Γ*-point. Thus, the mobility of both electrons and holes can be enhanced in tensile-strained GeNWs. The splitting valence bands also induce strong electron-phonon coupling and intraband scattering [33], which limits the hole mobility to be lower than the electron mobility.

## Conclusions

In summary, we have proposed tensile-strained GeNWs on GaSb, comparing three different situations via total energy change before and after Ge deposition. The result displays that the GeNW is inclined to form {105} surfaces along the 〈100〉 growth direction before the critical amount, while exposed by {110} surfaces after the critical amount. The residual strain field and bandgap analysis have shown that the same shape has the similar distribution both in strain and *Δ* *E*_DT_ regardless of size. Furthermore, the in-plane strain and the hydrostatic strain reduce not only from the edges to the center but also from the bottom to the top as well in all the situations. Due to the high tensile strain, almost the entire GeNW on GaSb can be converted into a direct bandgap material in the two possible situations. Also, the light holes mainly participate in the electron-hole recombination and electric transport at the *Γ*-point because the light hole band maxima becomes the maxima of the valence band in highly tensile strain. The mobility of not only electrons but also holes can be enhanced owing to the decrease of the carrier effective mass at the *Γ*-point determined by the tensile strain. The attractive performance predicted theoretically implies that tensile-strained GeNWs are promising to be applied to optoelectronics for light source and microelectronics for high speed devices in Si-photonics and electronics, respectively.
